# Phospholipase A and acyltransferase 4/retinoic acid receptor responder 3 at the intersection of tumor suppression and pathogen restriction

**DOI:** 10.3389/fimmu.2023.1107239

**Published:** 2023-03-31

**Authors:** Jian-Yong Zhao, Xiang-Kun Yuan, Rui-Zhen Luo, Li-Xin Wang, Wei Gu, Daisuke Yamane, Hui Feng

**Affiliations:** ^1^ Hospital of Integrated Traditional Chinese and Western Medicine, Hebei University of Chinese Medicine, Cangzhou, Hebei, China; ^2^ School of Medicine, Chongqing University, Chongqing, China; ^3^ Department of Diseases and Infection, Tokyo Metropolitan Institute of Medical Science, Tokyo, Japan

**Keywords:** retinoic acid, class II tumor suppressor, lipid metabolizing enzyme, p53, IRF1, interferon, infection

## Abstract

Phospholipase A and acyltransferase (PLAAT) 4 is a class II tumor suppressor with phospholipid metabolizing abilities. It was characterized in late 2000s, and has since been referred to as ‘tazarotene-induced gene 3’ (TIG3) or ‘retinoic acid receptor responder 3’ (RARRES3) as a key downstream effector of retinoic acid signaling. Two decades of research have revealed the complexity of its function and regulatory roles in suppressing tumorigenesis. However, more recent findings have also identified PLAAT4 as a key anti-microbial effector enzyme acting downstream of interferon regulatory factor 1 (IRF1) and interferons (IFNs), favoring protection from virus and parasite infections. Unveiling the molecular mechanisms underlying its action may thus open new therapeutic avenues for the treatment of both cancer and infectious diseases. Herein, we aim to summarize a brief history of PLAAT4 discovery, its transcriptional regulation, and the potential mechanisms in tumor prevention and anti-pathogen defense, and discuss potential future directions of PLAAT4 research toward the development of therapeutic approaches targeting this enzyme with pleiotropic functions.

## Introduction

Vitamin A (retinol), an essential fat-soluble nutrient widely present in the diet, is vital for human health and embryonic development (1, 2). The metabolism and biological functions of vitamin A have long been studied, and even its anti-carcinogenic effect in columnar mucous epithelium of the respiratory tract was reported over half a century ago (3, 4). This led to an outburst of research enthusiasm in understanding the anti-cancer activities of retinoic acid (RA, one of the biologically active metabolites of vitamin A) and its analogs (retinoids) in the 1970s and 1980s (5). Not surprisingly, numerous RA- and retinoid-inducible genes were identified and their functions have been characterized ever since, including tazarotene (a synthetic retinoid)-induced gene 1 (TIG1) and TIG2 (6–8).

TIG3, also referred to as RA receptor responder 3 (RARRES3), retinoid-inducible gene 1 (RIG1) or H-Ras-like suppressor 4 (HRASLS4), was first identified in 1998 from tazarotene-stimulated primary human keratinocytes (9). Given the long history of research aimed at uncovering the biological functions of vitamin A metabolites, it is not surprising to identify TIG3/RARRES3 as a class II tumor suppressor that mediates the anti-proliferative effects of retinoids soon after its discovery. Since then, numerous studies have reported the connection between reduced TIG3/RARRES3 expression and tumor progression (10–13). Notably, despite being identified as a tumor suppressor, later studies provided proof-of-principle evidence for the phospholipase A1/A2 and acyltransferase activities of TIG3/RARRES3 involved in phospholipid metabolism (14, 15). These findings led to a change in gene nomenclature from TIG3/RARRES3 to phospholipase A and acyltransferase 4 (PLAAT4) that reflects the enzymatic activities associated with the TIG3/RARRES3 protein. Although each of these alternative names reflects the RA-inducible nature of PLAAT4, they have caused confusions over its annotation. For instance, RIG1 was widely used to refer to DExD/H-box helicase 58 (DDX58, also known as RIG-I), the pattern recognition receptor (PRR) that senses pathogen-associated molecular patterns (PAMPs) and triggers the rapid activation of innate immune responses. Hence, we only use the official term PLAAT4 below for clarity.

Since its discovery, over two decades of research have unveiled the molecular mechanisms underlying PLAAT4-mediated restriction of tumor development and progression. More recently, the anti-pathogen properties of PLAAT4 have come to light, expanding its focus from cancer biology to anti-pathogen defense mechanisms (16, 17). In this review, we summarize recent progress in understanding how PLAAT4 promotes tumor suppression and pathogen restriction, focusing on its biochemical properties and transcriptional regulation, and discuss potential mechanisms in tumor prevention and anti-pathogen defense.

## PLAAT4 as a PLAAT family member

**Figure 1:**
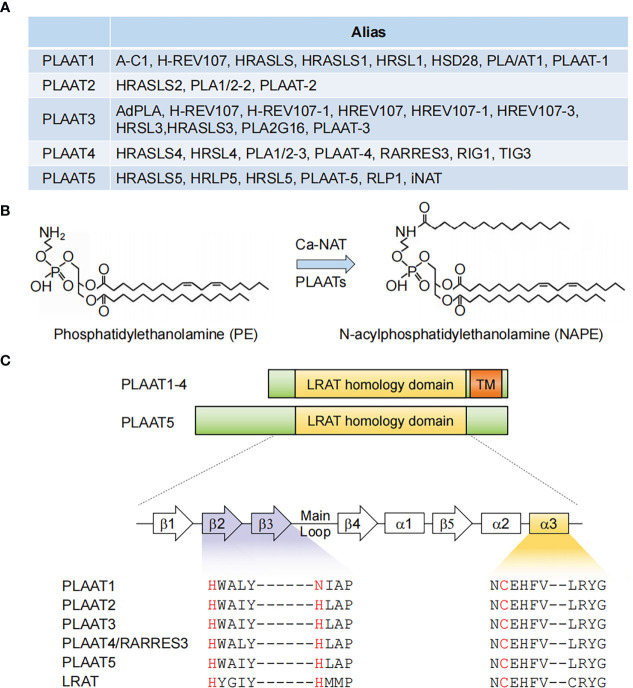
PLAAT family enzymes. **(A)** Gene names and aliases for all five PLAAT family proteins. **(B)** Illustration of an example of phospholipid metabolism reaction catalyzed by PLAAT family proteins. **(C)** Alignment of the structural domains of PLAAT family enzymes. The lecithin retinol acyltransferase (LRAT) homology and transmembrane (TM) domains are shown. The enzymatic activity of PLAAT proteins involves a cysteine-histidine-histidine triad, with the cysteine from a highly conserved ‘NCEHFV’ motif being the catalytically active site. α1−3, α helices 1-3; β1−5, β sheets 1-5.

In early studies, PLAAT4 was shown to share significant homology with another class II tumor suppressor − H-REV 107 (most well known as PLA2G16/HRASLS3/PLAAT3 among other names) (18, 19). These two members, together with HRASLS1, HRASLS2 and HRASLS5, which were all noted to be homologous to H-REV 107, are thus referred to as the H-REV 107 subfamily proteins or HRAS-like suppressors (14, 20, 21). Interestingly, frequent changes in nomenclature have similarly occurred to all these four latter members, leading to an incredibly large number of aliases in the literature (**Figure 1A**). This reflects struggles in understanding the biological nature of the H-REV 107 subfamily proteins.

In 2003, H-REV 107 was demonstrated to have originated from the lecithin retinol acyltransferase (LRAT) family, the representative member of vertebrate NlpC/P60 protein superfamily (22). Given that enzymes in this superfamily were known to be involved in phospholipid metabolism, the H-REV 107 subfamily proteins were subsequently identified to have *in vitro* Ca^2+^-independent phospholipase A1/2 and acyltransferase activities, and are thus capable of catalyzing several reactions in phospholipid metabolism (14, 21, 23, 24). For example, they have been shown to mediate the transfer of an acyl chain from glycerophospholipids, primarily phosphatidylcholine, to the amino group of the phosphatidylethanolamine, producing N-acylphosphatidylethanolamine (NAPE) that serves as the precursor for N-acylethanolamines (NAE) (**Figure 1B**). It should be noted, however, that this reaction was classically considered to be catalyzed by a Ca^2+^-dependent N-acyltransferase (Ca-NAT) (25–28). These seminal studies from the Ueda laboratory thus laid the foundation for renaming the H-REV 107 subfamily members as PLAAT1-5.

## Functional domains of PLAAT4

The catalytic activity of PLAAT4 depends on a 134 amino-acid hydrophilic region at the N-terminus. It shares several common features with LRAT as well as all other PLAATs, among which a highly conserved ‘NCEHFV’ motif is particularly prominent, with the conserved cysteine being identified as the active site catalytic nucleophile (**Figure 1C**) (15). According to comparative structural analysis of the catalytic domains from PLAAT2-4, the cysteine active site is placed in close proximity to the conserved histidine from β2 strand, thereby forming a cysteine-histidine-histidine catalytic triad that requires the histidine from β3 strand (15, 29, 30). Notably, this latter histidine is replaced by an asparagine residue in PLAAT1 (**Figure 1C**). Although it might still be positioned in a similar manner, it is tempting to anticipate that this subtle change may affect the substrate specificity and/or balance between the phospholipase A and acyltransferase activities of PLAAT1. Importantly, similar catalytic triad (i.e. cysteine-histidine-a polar residue) was also identified in the NlpC/P60 superfamily, indicating an evolutionary conserved attribute of these enzymes (22). In addition to the catalytic triad residues, structural evidence suggested that the highly flexible loop region between β3 and β4 strands adds fundamental alterations in the enzymatic activities of different PLAATs (30, 31). This is consistent with the structural context of the cysteine active site embedded in the hydrophobic pocket formed by the extended loops between strands β1 and β2, β3 and β4, and the N-terminal α3 helix (15).

PLAAT4 has a 30 amino-acid hydrophobic transmembrane domain following the N-terminal catalytic domain (**Figure 1C**). It encodes a single transmembrane-spanning segment, thereby directing PLAAT4 to cell membranes including plasma membrane, endoplasmic reticulum and Golgi apparatus (32, 33). Despite little sequence homology, this feature is shared by PLAAT1-3 but not PLAAT5, whose enzymatic activity is detected mainly in the cytosolic fraction (**Figure 1C**) (21, 23). Moreover, this transmembrane domain is not just an anchor tethering PLAAT4 to distinct membrane compartments, but itself is indispensable for an optimal biological function (34). For instance, it was demonstrated that the pro-apoptotic and anti-cancer activities of PLAAT4 are attributed primarily to the Golgi- rather than the endoplasmic reticulum-associated protein form (33). PLAAT4 at the plasma membrane, by contrast, was shown to facilitate the terminal stages in keratinocyte differentiation through interacting with type I transglutaminase (32, 35, 36).

Apart from the membrane system, PLAAT4 also distributes at the centrosome in skin cancer cells, leading to pericentrosomal organelle accumulation which in turn drives cancer cell apoptosis (24). A 24-amino acid segment (amino acids 102-125) that spans the β-sheet and α-helix immediately upstream of the hydrophobic tail was shown to be critical for the centrosome-targeting of PLAAT4 (37, 38). While the molecular mechanism underlying PLAAT4 translocation remains to be elucidated, a recent study showed that both zebrafish Plaat1 and murine PLAAT3 in the eye lens translocate from the cytosol to diverse organelles, which ultimately induces complete degradation of organelle membranes to achieve an optimal transparency of the lens (39). Likewise, cytosolic PLAAT3 in Hela cells can also translocate to endo-lysosomes following picornavirus infection, facilitating genome delivery into the cytoplasm from the micropores in endo-lysosomal membranes (40). Intriguingly, the translocation of Plaat1 and PLAAT3 both requires their C-terminal hydrophobic domain and is triggered by the membrane damage in organelle membranes. Therefore, it is highly plausible that both the hydrophobic tail itself and the upstream short-segment could serve as the recruitment cues, directing PLAAT4 towards target organelles where it acts as an acyltransferase and associates with other proteins for initiating signaling cascades.

## Transcriptional regulation of *PLAAT4*

**Figure 2:**
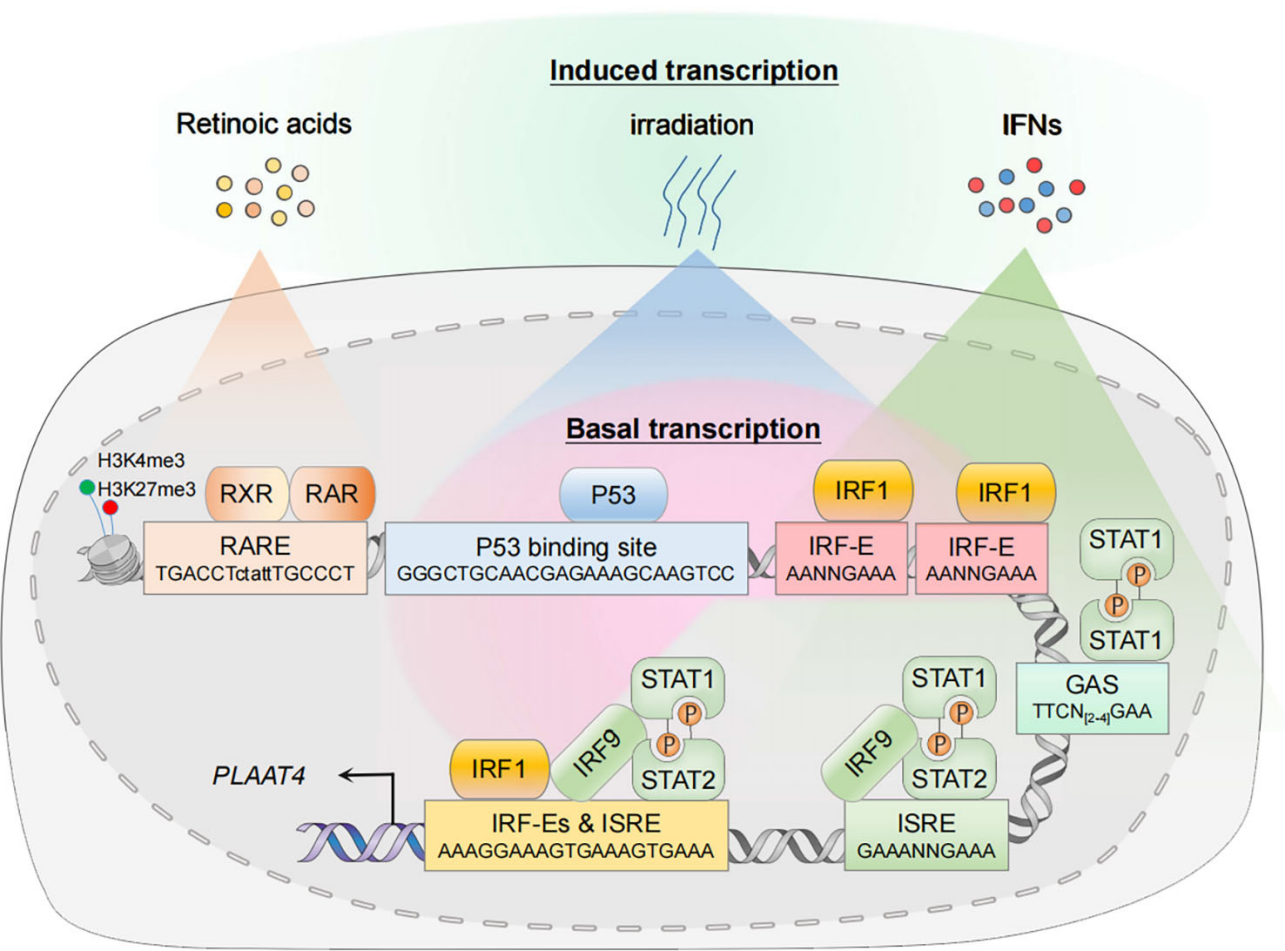
Transcriptional regulation of *PLAAT4*. In the resting state, nuclear transcription factors p53 and IRF1 mediate constitutive expression of *PLAAT4*. In the presence of agonists or acute stress, the corresponding signal transduction pathways are activated, thus leading to an enhanced *PLAAT4* transcription. Epigenetic modifications such as histone methylation also contribute to dynamic regulation of chromatin states, affecting binding of the RXR-RAR heterodimer and other transcription factors. RXR, retinoid X receptor; RAR, retinoic acid receptor; RARE, retinoic acid response element; p53-RE, p53 response element; IRF-E, IRF-binding element; ISRE, IFN-stimulated response element; GAS, gamma-activated sequence.

The *PLAAT4* gene is expressed ubiquitously in human cells, implying a tissue-wide protective role of its product. In addition, *PLAAT4* transcription is readily induced by retinoic acids and its natural and synthetic analogues, DNA-damaging stimuli, as well as interferons (IFNs), the secreted cytokines that orchestrate the induction of IFN-stimulated genes (ISGs) to establish an anti-pathogen state (discussed below) (16, 41, 42). Whether basally expressed or induced in response to different stimuli, the transcriptional activation of *PLAAT4* involves a high degree of selectivity. It is tightly controlled by transcription factors that specifically recognize and bind to functional *cis*-elements in its promoter, and this is essential for understanding the biological importance and complexity of PLAAT4 during pathogenesis (**Figure 2**). Hence, we summarize how transcription of *PLAAT4* is regulated under different biological contexts in the following subsections.

## Basal *PLAAT4* expression and its repression during tumorigenesis

The *PLAAT4* promoter contains the binding sites for transcription factors that constitutively activate *PLAAT4* transcription to exert a protective role at the basal level (16, 43). For example, a functional p53 response element (p53RE, -5157 to -5134 relative to the translation start site) exists in the *PLAAT4* promoter (44). Hence it is not surprising that p53, one of the best-characterized tumor suppressors expressed constitutively at low basal levels in unstressed cells, has the capacity to mediate *PLAAT4* promoter activation. Apart from p53, interferon regulatory factor 1 (IRF1) is also present at low basal levels in the nucleus where it manifests anti-oncogenic and anti-pathogen activities through maintaining optimal constitutive expression of hundreds of target genes, including *PLAAT4* (16, 45). Indeed, the *PLAAT4* promoter contains several IRF-binding elements (IRF-E), the hexanucleotide units with a core ‘GAAA’ sequence which are recognized specifically by the N-terminal DNA-binding domains from all IRF family members (46–48). However, it remains to be determined which IRF-E(s) are critical for IRF1-mediated *PLAAT4* transcriptional activation.

It has long been noted that at the heart of tumorigenesis and progression lies genetic mutations disrupting the harmonious checks and balances of normal cellular growth and development. In this context, *TP53* (encoding wild-type p53) is among the most frequently mutated genes in cancer, and the majority of tumor-derived mutations occurs in the region encoding DNA binding domain of p53 (49). This results in a diminished or even completely abolished activity to transactivate p53 target genes, including class II tumor suppressors like *CAV1*, *ING1b*, *SERPINB5*, etc. (50–54). Likewise, it was shown that a common abnormality in human leukemia and myelodysplasia is an interstitial deletion mapped to chromosome 5q.31, and *IRF1* is the only gene deleted consistently at one or both alleles within this region (55). Consistently, a decrease in *PLAAT4* transcript levels has been widely noted in various types of tumors (11, 12, 56–58). It is noteworthy that the loss of *IRF1* alleles *per se* does not lead to spontaneous tumor development (59). Rather, it favors a dramatic exacerbation of pre-existing genetic predispositions associated with other risk factors (e.g. *TP53* nullizygosity). This does not necessarily suggest that p53 plays a dominant role in driving *PLAAT4* transcription. In fact, PLAAT4 is abundantly expressed in T antigen-transformed cell lines in which p53 function is inactivated. Hence, while both IRF1 and p53 are key regulators of *PLAAT4* transcription, p53 likely activates a much broader anti-tumor transcriptional spectrum than IRF1.

## RA- and retinoid-induced *PLAAT4* expression and its regulation

Expression of *PLAAT4* is markedly induced by RA and its analogues, the biological effects of which are transduced through RA receptors (RARs) and retinoid X receptors (RXRs) as RAR-RXR heterodimers (60–63). They belong to the superfamily of nuclear receptors with ligand-dependent transcriptional activities, undergoing a shift from the repressive unliganded state towards its liganded active form that involves the exchange of co-repressors for co-activators, and could then drive the transcription of target genes *via* binding to a series of RA response elements (RAREs) that typically consist of hexameric direct repeats of (A/G)G(T/G)TCA with either a two (DR2) or five (DR5) nucleotide spacer (64–67). In agreement with the observation that *PLAAT4* transcription is enhanced by both RAR- and RXR-selective agonists, a functional DR5-type RARE was identified in the *PLAAT4* promoter (-5259 to -5243 relative to the translational start point) (10, 41). Interestingly, although this RARE is a non-canonical DR5-type element, it contains several conserved nucleotides which could form sequence-specific and water-mediated base contacts with residues Lys156 and Arg161 for RXR, and Lys109 for RAR (64, 68).

## *PLAAT4* expression in response to cellular DNA damage

In addition to being induced by RA and its analogs, it was shown that the expression of *PLAAT4* transcript and its protein product in human hepatoma HepG2 cells is robustly stimulated by DNA-damaging agents including 5-fluorouracil and UV irradiation (44). This is not surprising given that p53 and IRF1, besides acting at low basal levels, have been known as central hubs whose expression (i.e. transcription-coupled translation) is induced rapidly by various types of cellular stressors, resulting in either the repair or elimination of damaged cells by activating the expression of target genes (69, 70). DNA damage-induced PLAAT4 response is also consistent with the finding that tumor suppressor genes are activated prior to apoptosis effector genes as the levels of these two transcriptional factors increase (71). Interestingly, however, the PLAAT4 expression in HepG2 cells is not triggered by 2,3,7,8-Tetrachlorodibenzo-p-dioxin (TCDD), a persistent and ubiquitous environmental contaminant that induces oxidative stress and DNA damage (44, 72). This may be due to the counteraction effect of TCDD on p53 response *via* enhancing the expression of many other genes (73, 74). For example, TCDD was shown to enhance the protein levels of murine double minute-2 (MDM2), a multifunctional E3 ubiquitin ligase which specifically counteracts p53 function by binding to its transcriptional activation domain, as well as by targeting p53 for ubiquitination and subsequent proteasome-dependent degradation (75–78). In fact, a notable property of p53 and IRF1 is their flexibility to act coordinately with a variety of other co-factors (p63, RELA, etc.), allowing for a dynamic fine-tuning of target gene expression (79–82). Importantly, although p53 and IRF1 tend to act cooperatively in driving the expression of common downstream targets under such circumstance (e.g. *CDKN1A*, *a.k.a. P21*), it should be noted that p53 plays an absolutely dominant role in activating *PLAAT4* transcription in HepG2 cells treated with DNA damage agents (44, 83, 84).

## *PLAAT4* expression during host anti-pathogen responses

Being originally identified as a tumor suppressor, it is somewhat surprising that PLAAT4 is also one of the common downstream targets of IRF1 and IFN signals (16, 17, 85). IFN-dependent innate immunity is critical for host defense against invading pathogens. Cells engaged in such immune responses undergo sophisticated signal transduction that originates at the recognition of PAMPs by PRRs, leading to a rapid transcriptional activation of different types of IFNs and inflammatory cytokines. Secreted IFNs then initiate downstream Janus kinase (JAK)/signal transducer and activator of transcription (STAT) signaling *via* their cognate receptors, resulting in a second wave of transcriptional induction of hundreds of IFN-stimulated genes (ISGs) that depend mainly on the specific binding of IFN-stimulated gene factor 3 (ISGF3) heterotrimeric complex and STAT1 homodimer (known as IFN gamma activating factor; i.e. GAF) to IFN-stimulated response element (ISRE) and gamma-activated sequence (GAS), respectively (86–88). In agreement with the findings that *PLAAT4* functions as an ISG, both of these *cis*-elements exist in the *PLAAT4* promoter (**Figure 2**) (17). Notably, IRF1 was initially identified from crude nuclear extracts of Newcastle disease virus-infected mouse L929 cells, where it strongly activates transcription of the genes encoding type I IFNs (89–92). Moreover, IRF1 itself is also highly responsive to IFNs, especially type II IFN (IFNγ) (42). In this regard, IRF1 can induce much more robust and long-lasting expression of PLAAT4 (IFN-independent and IFNγ-dependent) than any other types of IFN during host innate immunity. Given that IRF1 can act in concert with STAT1 to induce ISG expression in IFN-stimulated cells, it is also reasonable to anticipate an enhancement of IFN-dependent *PLAAT4* expression by additional IRF1 binding (93, 94).

In addition to the classical role in cellular stress response, it is now widely appreciated that p53 plays essential roles during pathogen infections, in either an IFN-dependent or independent manner (95–97). Accordingly, pathogens have evolved sophisticated strategies to manipulate the p53 checkpoint for their own advantage. For instance, inhibition of p53 has been widely observed in the realm of viruses, bacteria as well as parasites (96, 98–101). While many studies are conducted in a p53-deficient background using the T antigen-immortalized and cancer cell lines, it is plausible to infer that *PLAA4* transcription may also be affected indirectly *via* p53 inhibition during pathogen infection.

## Mechanisms of PLAAT4 as a tumor suppressor

**Figure 3:**
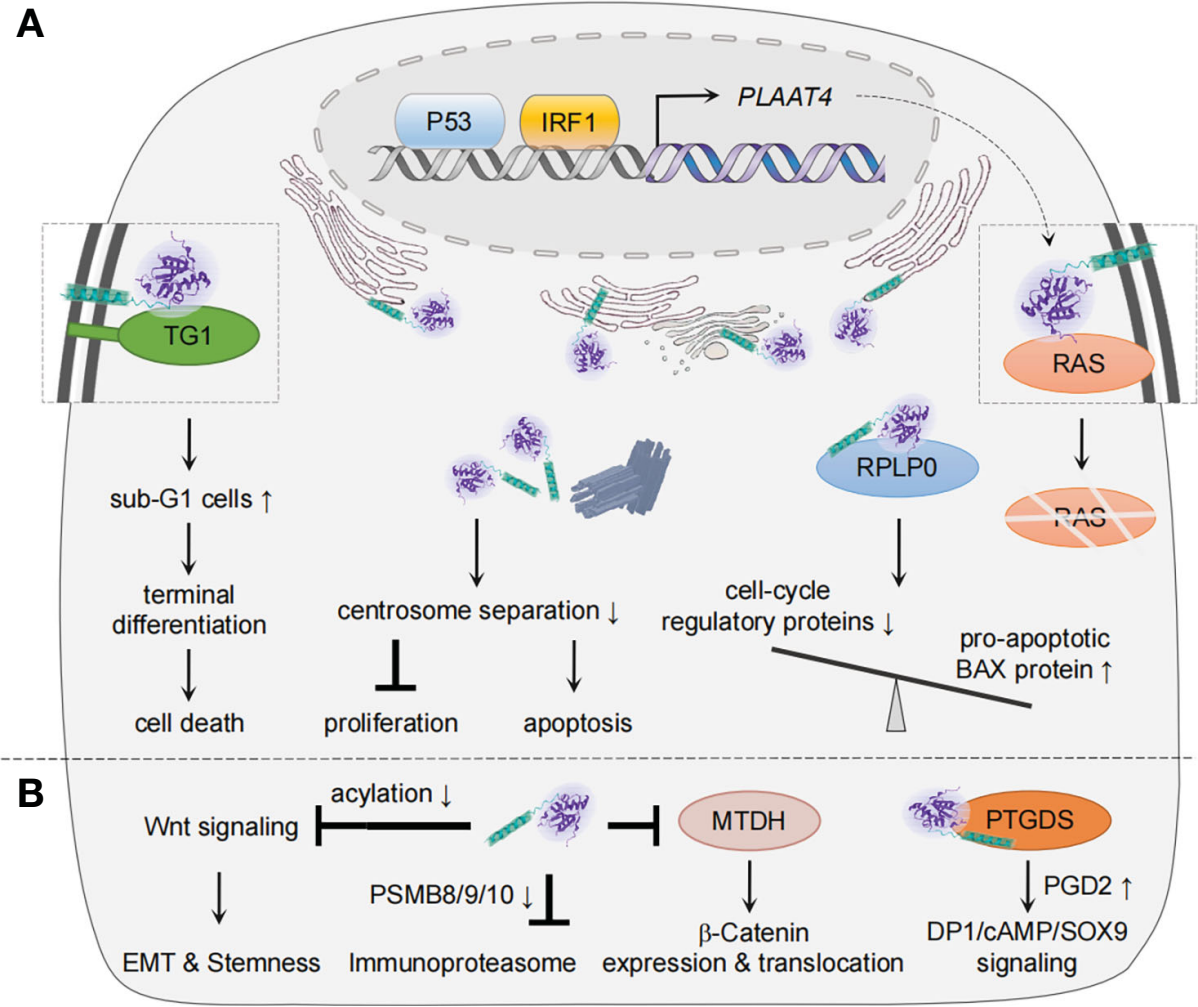
Mechanisms of PLAAT4 action during tumorigenesis and metastasis. **(A)** Nuclear p53 and IRF1 drive the basal expression of PLAAT4, which acts to regulate differentiation, proliferation, apoptosis, etc. to suppress tumorigenesis. The subcellular locations of PLAAT4 are shown, with a PLAAT4 structure predicted with alphaFold (105, 106). **(B)** PLAAT4 contributes to inhibition of epithelial-mesenchymal transition (EMT), cancer cell stemness, immunoproteasome, and β-Catenin expression and translocation, but induces prostaglandin D2 (PGD2)-mediated activation of DP1/cAMP/SOX9 signaling to suppress metastasis. For protein interactions where the subcellular location has not been identified, the illustration only depicts a physical interaction between PLAAT4 and target proteins.

As a class II tumor suppressor, PLAAT4 that is found abundantly in normal tissues has been noted to favor differentiation and apoptosis but inhibit cell proliferation and attenuates tumor growth (9, 32, 33, 102–104). As our understanding of the tumorigenic process has grown significantly, many mechanistic aspects of the tumor-suppressing activity of PLAAT4 have become clear during the past two decades (**Figure 3A**). In normal keratinocyte, for example, PLAAT4 interacts with type I transglutaminase at the plasma membrane, resulting in a substantial increase in the proportion of sub-G1 cells as well as nuclear shrinkage, hence forming unique structures that resemble the cornified envelope close to the cell surface (32, 35, 107). It then suppresses cell proliferation and induces a shift toward terminal keratinocyte differentiation, the ultimate outcome of which is cell death (108). Follow-up studies with epidermal squamous cancer cells suggested that PLAAT4 is also important for limiting cancer cell proliferation. Notably, PLAAT4 in these cells is largely localized near the centrosome, and thus could inhibit centrosome separation during mitosis, leading to the induction of cancer cell apoptosis (37, 109). These observations indicate that the expression levels of PLAAT4 are the determinant for modulating the balance between cell proliferation and survival/death. However, it should be noted that, while PLAAT4 promotes normal keratinocyte death *via* enhancing transglutaminase activity, the pericentrosomal localization of PLAAT4 in cancer cells drives organelle accumulation which in turn triggers caspase-dependent apoptosis (32, 109).

Interestingly, PLAAT4 appears to inhibit proliferation and promote apoptosis *via* different mechanisms in other types of cancer cells. For instance, it was suggested that PLAAT4 could form a protein complex with the ribosomal protein P0 (RPLP0) in HtTA cervical cancer cells (110). This is associated with a decreased RPLP0 level, resulting in a reduction in cell-cycle regulatory proteins but an enhancement in the pro-apoptotic BAX. PLAAT4 in HtTA cervical cancer cells was also shown to interact with the RAS, a plasma membrane-associated GTPase that regulates proliferation, differentiation and apoptosis by serving as binary switches (33, 111–113). The association between PLAAT4 and RAS not just alters the subcellular distribution of RAS, but also facilitates its degradation, thus leading to a decreased activation of RAS and downstream signaling. A recent study performed in a hepatocellular carcinoma model further supported this notion (58).

Besides being identified as a tumor suppressor, there is growing evidence suggesting that PLAAT4 also plays crucial roles in restricting tumor metastasis, the main cause of cancer-related death (**Figure 3B**) (13, 114–116). In this regard, *PLAAT4* is among the metastasis-associated gene signatures whose expression levels in primary breast tumors inversely correlate with the frequency of lung metastasis (117). Follow-up studies not only confirmed this observation, but unveiled the functional role of the catalytic activity of PLAAT4 in suppressing the lung metastasis (13, 118). Interestingly, while PLAAT4 dampens initial steps in the lung colonization through enforcing the retention of a phospholipase A1/2 activity-dependent differentiation features, it could also serve as an acyl protein thioesterase that hydrolyzes the acyl chains of Wnt proteins and a co-receptor in the canonical Wnt signaling to induce low density lipoprotein receptor protein 6 (LRP6). This contributes to the blockade of Wnt/β-catenin signaling, whereby suppressing epithelial-mesenchymal transition (EMT) and stem cell properties of tumor cells (114, 118). It is also noteworthy that PLAAT4 is expressed at significantly lower levels in steroid hormone receptors-positive (estrogen receptor-, progesterone receptor- and estrogen/progesterone receptors-positive (ER^+^, PR^+^ and ER^+^/PR^+^)) tissues than in ER-, PR- and ER/PR-negative tissues, hence providing a biomarker to identify a subgroup of patients with higher susceptibility to lung metastasis (103). In addition, a study reported the inhibitory effect of endogenous PLAAT4 on expression of the immunoproteasome subunits (PSMB8/9/10) in breast cancer cells (116). Although immunoproteasomes are most well-known for its role in antigen presentation, knocking down expression of its core subunit PSMB8 profoundly inhibits the migration and invasion of tumor cells (119–121). Hence, PLAAT4 could also indirectly suppress the distant metastasis of breast tumor cells by down-regulating PSMB8/9/10 expression (116).

In addition to the role in inhibiting breast tumor metastasis, PLAAT4 was shown to be capable of sequestering the oncoprotein metadherin (MTDH, *a.k.a.* AEG-1) that is involved in the development of various types of tumors, preventing MTDH from activating the cytoplasm-nuclear translocation of β-Catenin, whereby leading to suppression of the metastasis of colorectal cancer (115, 122, 123). The role of PLAAT4 in mediating cell migration and invasion was similarly demonstrated in NT2/D1 testicular cancer cells (124). Mechanistically, PLAAT4 physically interacts with glycoprotein prostaglandin D2 synthase (PTGDS, *a.k.a.* L-PGDS), a member of the lipocalin superfamily that has been shown to be involved in the tumorigenesis of solid tumors (125). It then promotes prostaglandin D2 (PGD2) production, resulting in the activation of PGD2 DP1 receptor/cAMP/SOX9 signaling. Notably, although an intact hydrophobic domain of PLAAT4 is critical for its interaction with PTGDS, the exact location for PLAAT4-PTGDS interaction remains unknown. It is interesting to note that H-REV107/PLA2G16/PLAAT3, a representative member of the PLAAT family, also inhibits migration and invasion of NT2/D1 testicular cancer cells by targeting at PTGDS (126).

Although a significant number of studies mentioned above have demonstrated PLAAT4 as a tumor suppressor, it remains to be determined whether its action generally requires the catalytic activity of PLAAT4, and how its catalytic products regulate the cellular events. Moreover, results from a recent study highlighted a positive correlation between PLAAT4 expression and the glioma grade, indicating its potential as a prognostic marker for poor survival (127). Molecular mechanisms responsible for this paradox remain unexplained, but such a phenomenon was also observed within the tumor microenvironment (a major barrier to immunotherapy) that induces the production of RA (128, 129). Despite being long considered as an anti-cancer agent, RA in solid tumors was found to display tumorigenic capability *via* myeloid-mediated immune suppression. Mechanistically, it polarizes intratumoral monocyte differentiation away from immunostimulatory dendritic cells but toward tumor-associated macrophages through suppressing dendritic cell-promoting transcription factor IRF4 (129).

## Mechanisms of PLAAT4 during anti-pathogen restriction

**Figure 4:**
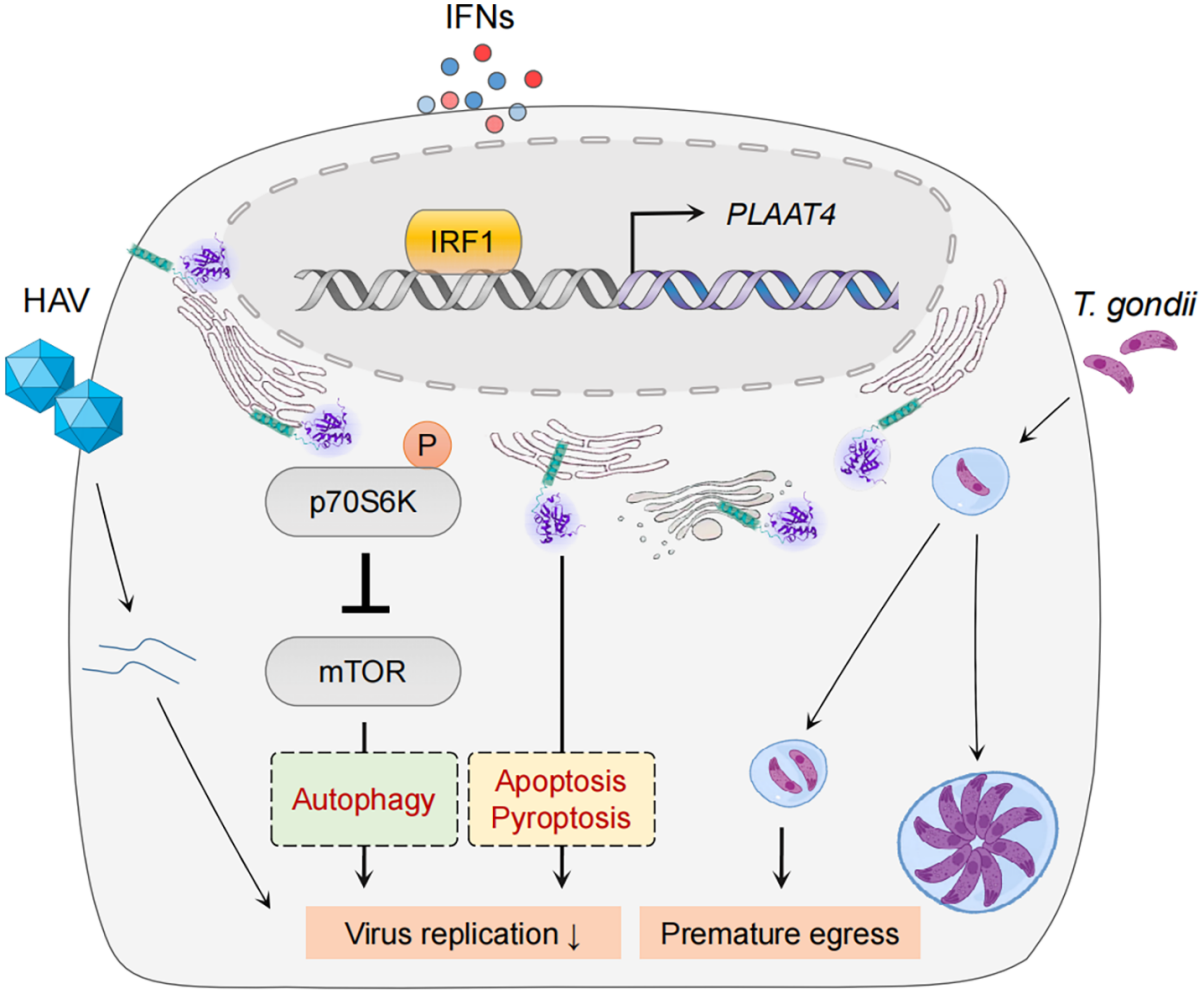
PLAAT4-mediated pathogen restriction. IRF1 and IFNs mediate expression of PLAAT4, which acts through in part by inactivating the mTOR signaling pathway to restrict hepatitis A virus replication (left), and induces premature egress to combat *Toxoplasma gondii* infection (right). PLAAT4-dependent resistance to viral replication might involve autophagy, pyroptosis and apoptosis (shown in dashed boxes).

Despite the fact that IFN and IRF1 pathways have long been considered to be highly effective at resisting and controlling pathogen infections, their common target PLAAT4 has not been recognized as a restriction factor for any pathogen until recently (**Figure 4**) (45, 130–133). For example, *PLAAT4* was identified as the most downregulated gene in IRF1-depleted human hepatocytes, and the most active one in limiting replication of hepatitis A virus (HAV), a notoriously stealthy picornavirus causing acute hepatitis in humans (16, 92, 134). Nevertheless, this robust antiviral activity of PLAAT4 seems virus-specific, as knocking down its expression only resulted in a modest (2- and 5-fold) enhancement in replication of dengue and Zika viruses (mosquito-borne flaviviruses causing dengue fever and Zika virus disease), and had no impact on replication of human rhinovirus (a picornavirus causing a variety of respiratory diseases) and hepatitis C virus (a hepatotropic flavivirus causing chronic hepatitis) (16). Likewise, PLAAT4 was also identified as an ISG that induces premature egress by reducing parasitic vacuole size, and thus actively restricts infection of the type III but not type I/II strains of *Toxoplasma gondii*, a parasite of warm-blooded animals that infects an estimated one-third of people worldwide (17, 135). Seemingly paradoxically, PLAAT4 as an effector of IRF1/IFNs does not affect or depend on the canonical innate immune signaling; rather, it directly restricts pathogen infections *via* the acyltransferase activity in both cases (16, 17). Considering the importance of innate immune responses in fighting against pathogen invasion, this may help to explain, at least partially, why anti-pathogen activity of PLAAT4 has long been neglected and why it is evident in a limited number of pathogens, especially those associated with mild or asymptomatic disease (e.g. HAV and type III *T. gonidii*) (134, 136). The anti-pathogen activity of PLAAT4 might also be related to degradation of specific host membranes through its phospholipase activity, a function that resembles PLAAT3 in the eye lens (39). Thus, identifying the selectivity of PLAAT4 would reveal the type of cellular organelle membranes specifically targeted by different pathogens for productive infection.

It is currently unknown the detailed mechanisms underlying anti-pathogen activity of PLAAT4. Nevertheless, the catalytic activity of PLAAT4 mediates induction of p70 S6 kinase phosphorylation at Thr389 and subsequent phosphorylation of mechanistic/mammalian target of rapamycin (mTOR) at Ser2448 that inactivates mTOR activity to phosphorylate 4E-BP1 in immortalized human hepatocytes (16). The functional importance of mTOR inhibition in restricting HAV replication was further confirmed by the observation that mTOR inhibitors fully recapitulate the HAV-specific suppression by PLAAT4 (16). Since pharmacologic mTOR inhibition in various cell types induces autophagy, a major digestion process that removes damaged (in this case infected) substrates, it is possible that PLAAT4 suppresses HAV replication indirectly through autophagy (137–139). Interestingly, results from a recent study confirmed that activation of mTOR signaling and in turn a suppressed autophagy are required for Zika virus replication (140). Therefore, our observations regarding the role of PLAAT4 in restricting replication of Zika virus might also be due to its ability to induce mTOR-dependent autophagy. However, it is important to note that Zika virus NS4A and NS4B proteins cooperatively suppress the mTOR pathway (141). It thus is tempting to speculate that Zika virus might have evolved to overcome mTOR-dependent autophagy inhibition; and consequently, this might help explain the limited anti-pathogen activity of PLAAT4 (142). For instance, mTOR as one of the most important regulators plays a significant role in orchestrating several cell processes including apoptosis (143). Notably, pathogen infections could directly stimulate mTOR activation, regulating expression of apoptosis-related proteins (e.g. inducing anti-apoptotic Bcl2 but suppressing pro-apoptotic Bax proteins), and then leading to the survival of infected cells (144, 145). Hence, it is reasonable to anticipate that PLAAT4 is capable of counteracting such a process by inhibiting mTOR activation. In this context, further studies are needed to dissect whether PLAAT4, especially its catalytic activity, can regulate mTOR-dependent autophagy and/or apoptosis to restrict pathogen infections. Moreover, as we discussed earlier, PLAAT4 expression during pathogen infections is under the concerted control of p53, IRF1 and IFNs, which all display pleiotropic effects on diverse cellular pathways other than mTOR signaling. For example, they have been demonstrated to play an important role in inducing apoptosis and/or pyroptosis (146–152). While our study has shown that PLAAT4-mediated suppression of HAV does not involve cell death (16), it remains to be determined whether mTOR-independent signaling pathways (e.g. apoptosis and pyroptosis, etc) are involved in the restriction of other pathogens.

Intriguingly, it should be pointed out that PLAAT4 is not the only PLAAT family member that has been demonstrated to affect the virus life cycle. For example, two independent genome-wide screens have identified H-REV 107/PLA2G16/PLAAT3 as a pivotal host factor for picornaviruses including poliovirus, rhinovirus, enterovirus, encephalomyocarditis virus and Saffold virus (40, 153). Mechanistically, the catalytically active PLAAT3 functions at a post-entry step, competing with galectin-8 and thus facilitating the delivery of viral genome to the cytoplasm which enables viral protein synthesis and subsequent RNA replication. Otherwise the virions will proceed to a pore-activated autophagic degradation pathway, enabling virus clearance procedure (40, 154). Besides serving as the switch between viral genome delivery and clearance, PLAAT3 also confers a selective cytotoxicity effect of the host cells exposed to rhinovirus infection, and this function depends on its C-terminal domain that extends into the endosomal lumen (153). In sharp contrast, PLAAT3 restricts hepatitis A virus infection instead of preventing viral genome from autophagy degradation (155). It is currently uncertain how PLAAT3 elicits opposing roles on different picornaviruses. However, it is important to note that despite HAV belongs to the *Picornaviridae* family, it distinguishes itself from other mammalian picornaviruses in the capsid structure, genome organization and replication cycle (156–159).

## Can PLAAT4 be an ideal therapeutic target?

A ubiquitous presence of PLAAT4 and its effector functions in tumor suppression and pathogen restriction suggest it might represent a self-protecting mechanism. In particular, PLAAT4 often declines in various types of tumor cells and tissues, but unlike the upstream transcriptional factors (e.g. p53 and IRF1), it is rarely mutated or deleted (9, 160). These characteristics of PLAAT4 make itself an attractive target for pharmacologic intervention to treat or palliate tumor-related symptoms, as well as to fight against pathogen infections.

In fact, the practical applications of vitamin A and its metabolites in anti-cancer therapy have been one of the most popular subjects (161–163). For example, all-trans retinoic acid (ATRA), the biologically active form of vitamin A and one of the first molecularly targeted drugs in oncology, has successfully eliminated fatal acute promyelocytic leukemia in cooperation with arsenic trioxide by targeting the prolyl isomerase Pin1 for degradation, and it is currently being evaluated for the feasibility in treating pancreatic cancer (164, 165). Notably, *PLAAT4* exhibits a time- and concentration-dependent expression pattern in response to ATRA, similar to IFNs that also play functional roles in anti-cancer immunity (10, 57, 166–168). Hence, therapies that target PLAAT4 for increased gene expression (e.g. uptake of vitamin A and its metabolites) might favor both pathogen restriction and tumor suppression, particularly for patients with p53- and/or IRF1-deficient cancers.

Being shared by anti-tumor and anti-pathogen responses, pathogen-triggered PLAAT4 expression may also offer potential to suppress tumorigenesis and development. This is reminiscent of applying microbial agents to treat malignant disease (169, 170). However, it is noteworthy that pathogenic microbial agents themselves are complicated, with some of them being well known to increase cancer incidence (e.g. human T lymphotropic virus type-1 oncovirus) (171, 172). Furthermore, although pathogen infections in general are expected to induce specific anti-tumor responses, a significant hurdle is that viral antigens often cause immunosuppressive effects that promote escape from the host immunity surveillance, thereby adding another layer of risk to the pathogen-triggered anti-tumor strategy (81, 92, 159, 171, 173). To bypass these impediments, live-attenuated or inactivated pathogens might be a suitable option for pathogen-initiated anti-tumor therapy (174–176).

It is important to note that PLAAT4 is just one of the hundreds of target genes induced by biological stimuli, and only a relatively small proportion of the gene products have been explored for their biological functions (133, 177–179). Although most of these effectors might not be potent enough to elicit tumor suppression and/or pathogen restriction when they act individually, they could mount an effective defense against a much wider spectrum of disease when induced as a combination with other effectors. This is exemplified by our findings that the antiviral effect of PLAAT4 is effectively amplified by contributions from other IRF1-effector gene products (e.g. NMI, MX1, ERAP2) with different mechanisms of action. However, the strategy to enhance the immune response is a double-edged sword that could potentially pose serious threats to the host if excessive and uncontrolled activation led to autoimmune-like disease. Thus, it would be necessary to identify other molecular targets that could act in synergy with PLAAT4 without adverse events associated with uncontrolled immune responses.

An alternative option would be to target specific expression of PLAAT4 or augment its phospholipase activity using small compounds, even though this sort of approaches may only partially restore the functions of its upstream agonists or transcriptional factors (16, 17, 57). Nonetheless, by identifying agonist-activated effectors that could target specific pathogens and cancers, host-directed therapeutic approaches that pinpoint such molecules would provide solid basis for more effective and safer therapies.

## Closing marks

As a member of the class II tumor suppressor gene family, PLAAT4 exhibits phospholipase A1/2 and acyltransferase activities with pleiotropic effects that profoundly affect host immunity against tumors and pathogens. As summarized in this review, substantial advances have been made in understanding the regulation of its expression, functional consequences and the mechanisms underlying its inhibitory effects on tumorigenesis and pathogens. It remains to be determined how PLAAT4 responses affect regulatory circuits with multiple feedback loops to control upstream signals involving RA metabolism and tumor suppressor (p53 and IRF1) and anti-pathogen (IFNs and IRF1) functions, thereby reinforcing both stress response and immunity. Given the numerous interacting proteins identified with PLAAT4, it could serve as a potential multiplex switch, fine-tuning key pathways to ensure an adequate response, or otherwise triggering signaling processes that favor apoptotic cell death.

Since it has just begun to appreciate the roles for PLAAT4 in the restriction of tumors and pathogen infections, several important gaps have remained in terms of mechanistic understanding of its function. For example, are there any other contexts where a *PLAAT4* transcription program is activated? What signals stimulate translocation of the membrane-localized PLAAT4 to other subcellular sites (e.g. centrosome) to transduce functional signals? What are the specific functions of endoplasmic reticulum-localized PLAAT4? Whether PLAAT4 regulates mTOR-dependent and independent autophagy and/or apoptosis in pathogen-infected cells? Understanding a comprehensive picture of PLAAT4 would open new avenues of PLAAT4 regulome that could impact biological events other than protection against cancers and pathogens. In addition to PLAAT4 itself, it would be important to clarify how its metabolite NAPE and changes in the host lipidome regulate the protein-protein interactions. It also remains to be determined whether PLAAT family proteins with similar biochemical features play redundant roles in protection from both invading pathogens and tumorigenesis. This is particularly important in understanding what cellular mechanisms may compensate for the lack of *PLAAT4* orthologs in rodents.

In summary, PLAAT4 research, while still in its infancy, has just begun to be emerged as an important field with relevance to host immunity to infection and disease progression. Although more investigations are required to answer above questions, further understanding of the molecular details of the PLAAT4 action would contribute to the development of more effective therapeutic approaches to treat tumors and pathogen infections.

## Author contributions

All authors contributed to conception of the topic. HF and J-YZ wrote the original paper and prepared the figures. DY provided intellectual input and wrote the paper. X-KY, R-ZL, L-XW and WG provided intellectual input. All authors contributed to the article and approved the submitted version.
